# Comparative Evaluation of the Placement of Drain Versus Normal Suturing to Eliminate Postoperative Complications After the Surgical Removal of Impacted Third Molar

**DOI:** 10.7759/cureus.51517

**Published:** 2024-01-02

**Authors:** Sarmatha Selvaraj, Indra Kumar Periyasamy, Gayathri P Elangovan, Arrvinthan Su, K. Srinath, B. Sivaragahvi

**Affiliations:** 1 Oral and Maxillofacial Surgery, Vivekanandha Dental College for Women, Tiruchengode, IND; 2 Periodontology, Vivekanandha Dental College for Women, Tiruchengode, IND

**Keywords:** sutures, trismus, flat drain, surgical drain, third molar extraction

## Abstract

Objective

This study aimed to compare the efficiency of corrugated rubber drains and sutures in preventing complications after the surgical removal of impacted mandibular molars.

Methodology

Sixty patients enrolled for surgical extraction of wisdom teeth were studied. The patients were divided into two groups of 30. After extraction, Group A received a corrugated flat rubber drain and Group B had only conventional knotted sutures in the extraction site. Patients were evaluated for postoperative pain, edema, and trismus. Before the procedure and on postoperative days 1, 2, and 7, all parameters were measured and compared.

Results

Patients in Group A with surgical drains showed a significant reduction in all postoperative challenges in contrast to Group B with normal sutures. The intergroup comparison indicates that pain was highest before surgery and showed a significant reduction by day 7 in both groups. Similarly, trismus was also at its peak before surgery for both groups. However, in contrast to Group A, Group B with suturing alone demonstrated a substantial reduction in trismus by day 7. By the end of day 7, edema had substantially decreased in both groups, but it was not statistically significant (*P* < 0.05).

Conclusions

The placement of surgical drains and the use of sutures alone have both shown similar and significant benefits in preventing postoperative challenges. However, intraoral drainage with a flat drain after mandibular third molar removal showed a significant reduction of pain, as measured by the visual analog scale (VAS) scale, or postoperative swelling.

## Introduction

Surgical extraction of impacted third molars is one of the most common surgical treatments provided by oral surgeons in dental clinics. Erupting third molars is the most common cause of pain, and it may be complicated by periapical inflammation, abscess, phlegm, or cyst [1]. Typical side effects experienced following extraction of an impacted tooth are reportedly pain, trismus, edema, and dry socket. Aside from the economic implications, the side effects of third molar surgery can result in significant morbidity, which may be severe enough to impede daily activities. These issues may be related to the type of suturing technique used depending on the length of the surgical procedure and the placement of intraoral drainage in the extraction site [2].

Partial wound closure, as opposed to complete wound closure, is used to alleviate pain, edema, and trismus following the extraction of the third molar [2]. The purpose of placing a drain after surgically extracting an impacted mandibular third molar is to provide a path for excessive exudate to flow out of the surgical site thus minimizing the possibility of subsequent hematoma formation [3]. Surgical drains are categorized as active or passive. Passive drains include tube drains, sheet drains, flat drains, open and closed drains, and external and internal drains [4]. For a variety of surgical procedures, including plastic and reconstructive surgery, flat drains are employed. Flat drains operate by the forces of gravity or changes in intracavity pressure. When the draining fluid is too viscous to move via tubes, these drains are utilized. There is less tissue damage, reduced wound infection, and no skin excoriation [1].

Thus, the goal of this study is to examine and compare the effectiveness of corrugated rubber drain and suturing in preventing post-operative complications following surgical removal of impacted mandibular molars.

## Materials and methods

**Figure 1:**
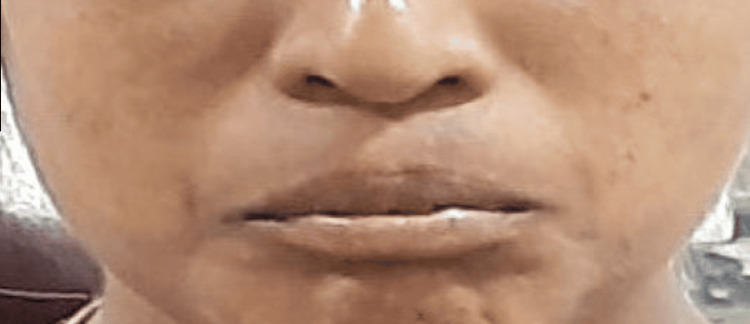
Preoperative image of a patient in Group A without any extra oral swelling.

**Figure 2:**
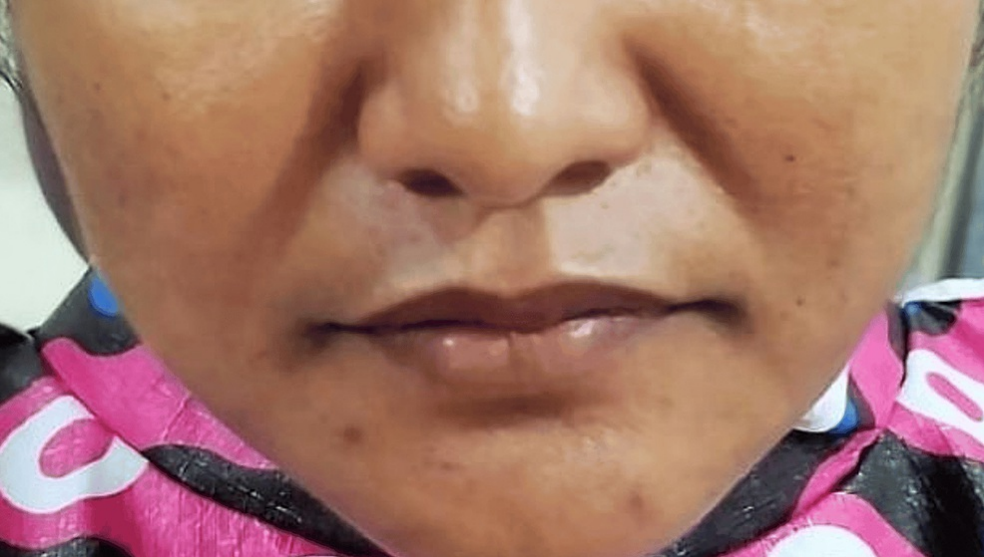
Preoperative image of a patient in Group B without any extra oral swelling.

The investigation was carried out at the Vivekanandha Dental College for Women (VDCW). Ethical committee approval (VDCW/IEC/309/2022) was obtained before the commencement of the study. The research group was chosen from individuals who visited the Department of Oral and Maxillofacial Surgery for third molar extractions. Sixty healthy adults without extraoral edema (Figures 1-2) were randomly assigned to two groups.

Each patient was given a thorough explanation of the procedure for surgery, and signed informed consent was obtained for their participation in the trial. Each group consisted of thirty patients. Group A participants received a surgical drain, and Group B subjects underwent standard suturing post-extraction. Based on Pederson's index [5], Groups A and B were further divided into three subgroups: (i) Subgroup A - very difficult (8-10); (ii) Subgroup B - moderately difficult (5-7); and (iii) Subgroup C - slightly difficult (3-4).

The inclusion criteria encompassed individuals with unilateral mandibular impacted molars, aged between 18 and 70, exhibiting no additional extraoral edema. Participants were enrolled and postoperatively monitored in adherence to the study protocol. Exclusion criteria comprised individuals under 18, pregnant women, those with any systemic illness, individuals lacking pericoronal inflammation, smokers, and those unwilling to participate in the trial were excluded. The visual analog scale (VAS), which ranges from 0 to 10, was used to quantify postoperative pain levels. The initial assessment was done immediately following surgery. An electronic caliper was used to assess the degree of mouth opening. The Sauza and Consone [6] analysis was utilized to determine the swelling. Horizontal measurements were recorded from the corner of the mouth to the point of connection of the ear lobe. By palpating and identifying the inferior boundary, the vertical distance from the outer canthus of the eye to the angle of the mandible was determined. All measurements were recorded before the surgery and on postoperative days 1, 2, and 7.

**Figure 3:**
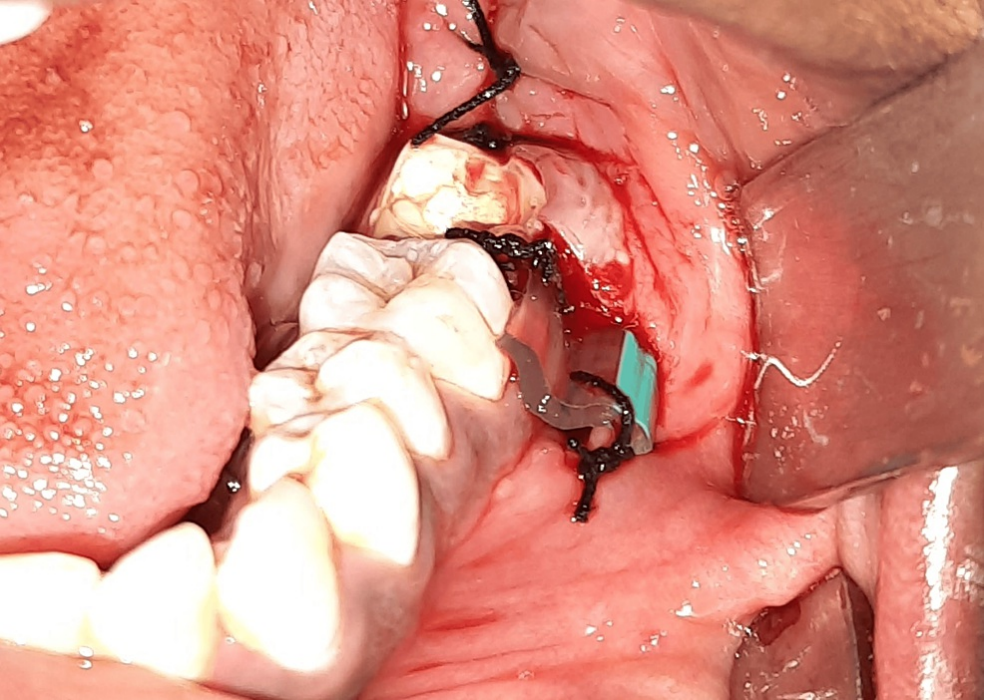
Postoperative image of a patient in Group A after the placement of a surgical drain.

Under local anesthesia, an oral and maxillofacial surgeon with extensive experience surgically removed the wisdom tooth. After the extraction, the tooth socket was rinsed and cleansed with saline, and any bony margins were subsequently removed. A corrugated rubber drain of 0.8 mm in thickness, 1.5-2 cm in width, and 5.5 cm in length was inserted into the floor of the oral vestibule close to the releasing incision for group A patients (Figure 3).

The size of the drain was modified to suit the drainage requirements. A single-knotted, non-resorbable 3-0 silk suture was used to reposition the mucoperiosteal flap and hold it in place. The drain was removed after 24 hours. For patients in Group B, suturing alone was performed. After surgery, each patient was given extensive guidance on how to care for the surgical site. In addition to taking 100 mg of Ketoprofen twice daily for three days, patients were instructed to rinse their mouths with a 0.1% chlorhexidine gluconate solution for the next seven days. On postoperative days 1, 2, and 7, the patients were recalled to record the clinical parameters.

IBM SPSS Statistics for Windows, Version 26.0 (IBM Corp., Armonk, NY) served as the tool for all statistical analyses. Results of descriptive statistics were presented as standard deviation and mean. Categorical variables were correlated using Fisher's exact test and the chi-square test. For intergroup comparison, the Mann-Whitney test was applied. The Cochran's Q test for related samples was employed to compare categorical variables within groups. Continuous data analysis was done using Friedman's repeated measures analysis of variance by ranks. Statistics were deemed to be significant at a *P*-value of <0.05.

## Results

The study consisted of 60 patients who were candidates for the surgical removal of their mandibular third molars. The patients were randomly divided into two groups, each comprising 30 individuals. Group A (*n *= 30) received a corrugated rubber drain; Group B (*n* = 30) received non-resorbable 3-0 silk sutures alone after extraction. Table 1 summarizes data before and after surgery. It shows stable VAS scores for pain, consistent trismus, and swelling levels. The *P*-value of 0.001 indicates significant changes in these variables after surgery. Additionally, dry socket incidence decreased significantly from 56.7% on day 1 to 0% on days 2 and 7, with a *P*-value of 0.001. These findings suggest that the surgery had a significant impact on the measured variables (Table 1).

**Table 1:** Comparison of variables before surgery and days 1, 2, and 7 in Group B. Cochran's Q test for related samples; Friedman's repeated measures analysis of variance by ranks; significant, *P* < 0.05. SD, standard deviation; VAS, visual analog scale

Variables	Before surgery	Day 1	Day 2	Day 7	*P*-value
VAS scores					
Mean ± SD	3.2 ± 1.0	3.2 ± 1.0	3.2 ± 1.0	3.2 ± 1.0	0.001
Median	3.0	3.0	3.0	3.0	
Trismus					
Mean ± SD	39.1 ± 2.2	39.1 ± 2.2	39.1 ± 2.2	39.1 ± 2.2	0.001
Median	39.5	39.5	39.5	39.5	
Swelling					
Mean ± SD	10.1 ± 0.5	10.1 ± 0.5	10.1 ± 0.5	10.1 ± 0.5	0.001
Median	9.9	9.9	9.9	9.9	
Dry socket					
*N* (frequency)	-	17 (56.7 %)	0	0	0.001

Patients in Group A with surgical drains showed a significant reduction in all postoperative challenges compared with Group B with normal sutures. The intergroup comparison indicates that pain was highest before surgery and showed a significant reduction by day 7 in both groups. The outcomes in both groups were analogous. Similarly, trismus was also at its peak before surgery for both groups (Group A, 38.0 ± 1.5; Group B, 39.1 ± 2.2). In comparison to Group B, which underwent suturing alone (30.6 ± 2.3), Group A, receiving both suturing and a surgical drain (36.0 ± 1.8), demonstrated a substantial improvement in mouth opening by day 7. By the end of day 7, edema had substantially decreased in both groups, but it was not statistically significant (*P* < 0.05). In Group A, from days 1 to 7, there was a notable decline in pain and edema, as evidenced by data from the intragroup comparison of Group A. The mouth opening score on day 1 was 26.5 ± 3.4, but it gradually improved by the end of day 7 (36.0 ± 1.8) (Table 2).

**Table 2:** Comparison of variables between groups. Mann-Whitney U test; significant, *P *< 0.05. SD, standard deviation; VAS, visual analog scale

Variables	Group A (with drain)		Group B (with suture alone)		
	Mean ± SD	Median	Mean ± SD	Median	*P*-value
VAS scores					
Before surgery	2.0 ± 1.1	2.0	3.2 ± 1.0	3.0	0.001
Day 1	1.1 ± 0.7	1.0	1.8 ± 0.8	2.0	0.002
Day 2	0.4 ± 0.5	0.0	1.2 ± 0.9	1.0	0.001
Day 7	0.1 ± 0.3	0.0	0.4 ± 0.5	0.0	0.004
Trismus					
Before surgery	38.0 ± 1.5	38.0	39.1 ± 2.2	39.5	0.001
Day 1	26.5 ± 3.4	26.6	22.4 ± 1.8	22.7	0.001
Day 2	31.3 ± 2.4	30.6	26.5 ± 2.0	26.7	0.001
Day 7	36.0 ± 1.8	35.5	30.6 ± 2.3	30.2	0.001
Swelling					
Before surgery	9.9 ± 0.4	9.8	10.1± 0.5	9.9	0.149
Day 1	9.8 ± 4.5	9.0	9.8 ± 3.2	10.0	0.712
Day 2	6.8 ± 3.9	6.3	6.9 ± 2.8	6.3	0.652
Day 7	4.3 ± 3.6	3.8	3.7 ± 2.5	3.1	0.762

The data suggest that the surgical procedure had a significant impact on reducing pain (VAS scores) and dry socket incidence but had no significant effect on trismus or swelling levels. The consistent *P*-values of 0.001 reinforce the statistical significance of these findings (Table 3).

**Table 3:** Comparison of variables before surgery and days 1, 2, and 7 in Group A. Cochran's Q test for related samples; Friedman's repeated measures analysis of variance by ranks; significant, *P* < 0.05. SD, standard deviation, VAS, visual analog scale

Variables	Before surgery	Day 1	Day 2	Day 7	*P*-value
VAS scores					
Mean ± SD	2.0 ± 1.1	1.1 ± 0.7	0.4 ± 0.5	0.1 ± 0.3	0.001
Median	2.0	1.0	1.0	1.0	
Trismus					
Mean ± SD	38.0 ± 1.5	38.0 ± 1.5	38.0 ± 1.5	38.0 ± 1.5	0.001
Median	38.0	38.0	38.0	38.0	
Swelling					
Mean ± SD	9.9 ± 0.4	9.9 ± 0.4	9.9 ± 0.4	9.9 ± 0.4	0.001
Median	9.8	9.8	9.8	9.8	
Dry socket					
*N* (frequency)	-	12 (40 %)	0	0	0.001

## Discussion

Third molar extraction frequently leads to postoperative discomfort, facial edema, and trismus; however, primary healing and the use of a surgical drain appear to be effective in minimizing these consequences. Numerous authors have investigated the usefulness of a surgical drain for dealing with postoperative comorbidities following third molar surgery. The least invasive drainage technique, i.e., the application of flat drains, was selected for our investigation. Flat drains can be used as an alternative to round drains to facilitate the free flow of exudate as they are less intrusive and more effective in minimizing postoperative effects. Due to the flat drain's near-invisibility in the mouth and ease of removal, patients do not experience as much discomfort. After surgery, knotted sutures are used to secure the extraction site, which lessens postoperative bleeding and helps avoid post-extraction alveolitis by trapping the clot within the alveolus [1]. Since sutures were used in both groups, neither group experienced a dry socket by the end of day 7.

According to research by Deliverska and Petkova, pain is worse just after surgery and steadily reduces over the subsequent days [7]. Both groups experienced a similar decrease in pain intensity during the evaluation period, suggesting that there was no discernible difference among them. Our findings are consistent with that of Rakprasitkul and Pairuchvej [8]. As reported by Seymour et al. [9], the pain experienced following the extraction of mandibular molars with only sutures is highest on the day of surgery and worsens up to 12 hours. This is consistent with the outcomes of our research. The group receiving only sutures had significantly higher mean VAS scores reported on the first day of surgery, which then declined by day 7. Following surgery, wound closure by primary intention was faster and preferable, but it caused increased edema, pain, and discomfort since exudate got pooled and exerted pressure on the nerves and blood vessels [10]. The installation of a surgical drain lessened the quantity of accumulated blood or serum from the socket, which may have aided in alleviating the discomfort experienced after surgery.

We discovered that the group with a drain and the group with only sutures showed gradual improvement in mouth opening from days 1 to 7, proving that there was no apparent benefit to employing the surgical drain in lowering the extent of trismus. Saglam [11] reported a similar outcome, claiming that surgical tube drainage decreased postoperative facial edema but had no appreciable impact on the severity of trismus. In our investigation, the primary wound closure was carried out using sutures after the drain was inserted into a releasing incision in the vestibule. This technique lowers the chance of major postoperative hemorrhage and trismus while also preventing the collection of food particles within the wound. Jaw opening was assessed before surgery as well as on postoperative days 1, 2, and 7. This was determined using an electronic caliper by measuring the maximum distance between the incisal edges of the central incisor in the maxilla and mandible. The least amount of jaw opening was observed after day 1 in all study groups. By day 7, the mouth opening had improved significantly in both groups, and this was in line with the research results of Cerqueira et al. [12] and Genc et al. [13].

Swelling, which starts gradually and reaches its peak within 48 hours following surgery, is primarily responsible for social isolation among many patients. After day 4, it gradually diminishes and completely subsides within seven days [14]. According to Hashemi et al. [15], after the extraction of impacted teeth, postoperative edema could be minimized by establishing a channel through which inflammatory fluids may be expelled from the surgical site. In the initial postoperative phase, our observations, however, showed no significant differences among the groups in terms of reduction of swelling. In our investigation, the most substantial swelling was detected in all study groups on day 1, and by day 7, the swelling was considerably reduced in both groups. However, in the surgical drain group, the extent of swelling was much lower on day 7 than in the group with sutures alone. The findings of our investigation were supported by studies by Kumar et al. [16] and Obimakinde et al. [17] that demonstrated the use of rubber drains in significantly minimizing postoperative facial inflammation.

The key points in the study reveal that third molar extraction is known to cause postoperative discomfort, facial swelling, and trismus. The study aimed to investigate the use of surgical drains to minimize these issues. The study utilized flat drains as an alternative to round drains, as they are less intrusive and more effective in minimizing postoperative effects. Flat drains are nearly invisible in the mouth and cause less discomfort during removal. Both study groups had sutures to secure the extraction site, reducing postoperative bleeding and preventing post-extraction alveolitis (dry socket) by keeping the clot in place. Pain was assessed in both groups, and it was found that pain was worse immediately after surgery but gradually decreased over the following days. The study suggests that there was no significant difference in pain reduction between the group with a surgical drain and the group with only sutures. Both groups showed a gradual improvement in mouth opening from days 1 to 7, indicating that the use of a surgical drain did not seem to provide a clear benefit in reducing trismus (difficulty in mouth opening). Swelling was observed to be at its peak within 48 hours after surgery and gradually decreased over time. The surgical drain group had lower swelling on day 7 compared to the group with sutures alone. In summary, the study suggests that the use of surgical drains may help reduce postoperative swelling and improve wound healing after a third molar extraction. However, there were limitations to the study that need to be considered, and further research is recommended to confirm these findings and provide a more comprehensive understanding of the benefits of surgical drains in this context.

The study has some limitations such as a relatively small sample size of 60 patients could limit the generalizability of the findings. Additionally, the short follow-up period, with evaluations conducted only on postoperative days 1 and 7, might not capture potential longer-term effects accurately. Additionally, the absence of a placebo or no-treatment group made it challenging to assess the true impact of each intervention. The absence of specific patient selection criteria and blinding procedures might introduce biases into the results. Moreover, relying on subjective measures like the VAS scale for pain assessment might be influenced by patients' perceptions and reporting bias. Addressing these limitations through larger, multicenter studies with longer follow-up periods and more rigorous methodology will enhance the reliability and applicability of the conclusions in clinical practice.

## Conclusions

Within the scope of our study, following the extraction of mandibular third molars, both the insertion of surgical drains and the placement of sutures alone have demonstrated similar and substantial benefits in the prevention of postoperative challenges. However, postsurgical edema, pain, and trismus were significantly reduced when a surgical flat corrugated rubber drain was used. Thus, traditional wound closure along with a drain could have an added advantage in alleviating postsurgical issues. Intraoral drainage with flat drains can hasten the patient's recovery, make it less traumatic, and expedite the patient's return to normal activities.
